# A Safety-Governed Architecture for Adaptive Sequential Evidence Selection from Pre-Recorded Gait Data

**DOI:** 10.3390/s26196120

**Published:** 2026-09-27

**Authors:** Giulio Leone, Daniela D’Auria

**Affiliations:** Department of Engineering and Sciences, Universitas Mercatorum, 00186 Rome, Italy; giulio.leone@studenti.unimercatorum.it

**Keywords:** sequential evidence selection, retrospective gait analysis, pre-recorded sensor data, multi-agent systems, expected information gain, safety governance, reproducibility

## Abstract

Sequential evidence selection can reveal which parts of an existing sensor record reduce model uncertainty, while providing a computational test bed for future adaptive sensing. This work introduces the Embodied Evidence Acquisition and Reasoning Loop (EARL), a typed architecture in which five role-specialized critics score evidence requests and a deterministic safety governor retains exclusive execution authority. Observed, derived, and simulated evidence remain provenance-distinct in a replayable, hash-linked ledger. The primary experiment sequentially disclosed precomputed feature bundles from pre-recorded gait data; it did not acquire new measurements. EARL was evaluated on 64 unique subjects from the PhysioNet Gait in Neurodegenerative Disease Database using repeated subject-level cross-validation, 11 predeclared conditions, and 3520 replay-verified runs. The primary endpoint was area under cumulative posterior-entropy reduction. EARL achieved 7.689 (95% CI 7.535–7.840), exceeding fixed-order and random selection by 0.459 and 0.763, respectively; the EARL-minus-EIG difference was –0.321. EARL had descriptively higher macro accuracy (55.8% versus 49.5%) and a lower Brier score (0.781 versus 0.813) than pure expected-information-gain selection. This measures an entropy-efficiency trade-off under additional selection criteria, not universal superiority. All 10 original software safety challenges produced their expected outcomes. Separately identified post hoc analyses examine probe use, a sensitivity analysis excluding the record-quality probe, critic influence, illustrative resource costs, early stopping, and safety-constrained simulated execution. The small retrospective cohort and illustrative simulations establish software behavior, not clinical diagnostic performance, treatment benefit, physical-robot safety, or patient efficacy.

## 1. Introduction

Sensor-rich rehabilitation environments create an opportunity to turn uncertainty into an explicit sequence of measurements. A wearable device or rehabilitation robot can expose timing, force, kinematic, physiological, and interaction signals; unlike a static clinical record, it can also support a protocol in which the next measurement is selected after observing the previous one. This distinction is particularly relevant when a presentation is uncommon, heterogeneous, or insufficiently explained by the evidence already available. The appropriate role of such a system is not to autonomously declare a diagnosis, but to organize testable hypotheses, select bounded evidence-acquisition actions, and present a traceable record for expert review.

Existing rehabilitation datasets demonstrate that movement sensing can quantify clinically relevant structure. Bilateral foot-force records capture altered gait dynamics in Huntington’s disease and amyotrophic lateral sclerosis (ALS) [1,2]; RGB-D and motion-capture datasets support exercise assessment [3,4]; and wearable algorithms have profiled gait and balance in the rare neuromuscular disorder Charcot–Marie–Tooth disease type 1A [5]. These studies establish measurement feasibility, but they do not define how an autonomous system should decide which evidence to obtain next or how such a decision should be constrained before a robot can act.

In parallel, agentic systems have advanced from document retrieval toward tool-using rare-disease decision support [6,7] and iterative scientific workflows [8,9]. Minimal experiment loops can repeatedly modify and retain computational experiments [10], while population-based agent simulation can explore many interacting scenarios [11]. Closed-loop laboratories further show that automated hypothesis–experiment feedback can produce transferable knowledge in chemistry [12]. Embodied rehabilitation is a stricter setting: an agent proposal may eventually reach a physical actuator near a person, so provenance, execution authority, idempotency, and fail-closed behavior are not optional implementation details.

We present EARL, the Embodied Evidence Acquisition and Reasoning Loop, as a safety-governed architecture for sequential sensor evidence acquisition under these constraints. EARL separates proposal authority from execution authority. Role-specific critics may rank a typed probe, and a simulator may estimate its information value, but only deterministic validation and a versioned safety envelope can approve an adapter call. Every accepted or rejected action is written to a content-addressed evidence chain. The reference implementation is model-agnostic at the agent boundary: the present evaluation uses deterministic critics so that system behavior can be reproduced exactly, while the same validated score-card contract can contain future language-model agents without granting them actuator authority.

This study makes the following four contributions:

1. A typed closed-loop architecture that keeps observed, derived, and simulated evidence distinct and makes the safety governor the sole execution authority.
2. A five-critic hypothesis council combining informativeness, falsification, diversity, feasibility, and safety assessments with explicit veto semantics.
3. A replayable implementation with strict contracts, idempotent adapter execution, deterministic safety challenges, and a parent-linked SHA-256 research ledger; and.
4. A leakage-controlled retrospective evaluation on 64 unique subjects, comprising 3520 subject-condition runs across comparators, ablations, and robustness scenarios.

The task evaluated here is adaptive selection of information from a pre-recorded database: after each disclosed feature bundle, the policy selects the next bundle using its updated posterior. This differs from adaptive physical acquisition, in which a new sensor measurement or task changes what data exist. The primary experiment tests only the former; a retrospective bundle is not an independently acquired measurement. Active responses are represented only by declared in-silico scenarios and software safety challenges. No participant was recruited, no physical robot was commanded, and no clinical decision was made. The study therefore does not demonstrate clinical diagnostic performance, therapeutic benefit, physical-robot safety, or patient outcomes. Rehabilitation sensing is a prospective application of the architecture, not an experimentally validated deployment.

## 2. Related Work

### 2.1. Rehabilitation and Rare-Disease Sensing

The PhysioNet Gait in Neurodegenerative Disease Database (GaitNDD) combines bilateral force-sensitive-resistor recordings and stride-derived series for ALS, Huntington’s disease, Parkinson’s disease, and control cohorts [13,14]. Its subject-level recordings make it suitable for evaluating sequential disclosure of gait evidence, provided that all learned quantities remain inside training folds. Other rehabilitation benchmarks broaden the sensing modalities: UI-PRMD combines optical motion capture and Kinect observations [3], while KIMORE links RGB-D exercise motion with clinical scores [4]. A recent therapist–exoskeleton–patient dataset includes encoders, interaction torque, electromyography, inertial measurements, and force signals during gait therapy [15]. These resources motivate a future multimodal adapter layer, but only GaitNDD is used in the present benchmark.

Rare-disease sensing and rare-disease reasoning should not be conflated. Disease-specific wearable models can characterize activity within a defined rare cohort [5]; conversely, recent agentic systems coordinate medical roles, tools, and traceable evidence for differential diagnosis [6,7]. Earlier clinical-computing work combined intelligent agents with complex event processing for patient monitoring, used entity linking to improve graph embeddings from Italian clinical notes, and detected expected activities from medical-simulator data stored in scientific databases [16,17,18]. EARL occupies the interface between these directions: it asks how a reasoning workflow could request the next bounded sensor observation. Its four cohort labels are an evaluation surrogate for uncertainty reduction, not claims that gait alone can diagnose these diseases.

### 2.2. Active Perception and Autonomous Science

Active perception frames sensing as an action chosen to improve task-relevant information [19]. EARL instantiates that principle with explicit probes, costs, risk classes, and posterior updates. Expected information gain (EIG) supplies one acquisition objective, but an embodied research action may also need to discriminate the leading alternatives, avoid redundant evidence, respect sensor availability, and satisfy a safety protocol. The council therefore treats information gain as one criterion rather than the entire objective.

Autonomous-science systems have implemented recurring cycles of hypothesis generation, experiment execution, analysis, and manuscript production [8,9]. The autoresearch software pattern emphasizes a simple measurable objective and a keep-or-discard loop [10]; MiroFish illustrates large-scale multi-agent scenario simulation [11]. EARL borrows the general principles of repeated measurable experiments and plural agent perspectives, but neither project is a software dependency and neither supplies biomedical safety evidence. Performance metrics and explicit autonomy levels are essential when evaluating self-driving laboratories [20], while human oversight remains a deliberate part of emerging closed-loop laboratory practice [21]. Controlled evidence also cautions that generative systems may fail at scientific hypothesis and experimental reasoning without grounded feedback [22]. These observations motivate EARL’s typed outputs, deterministic validation, evidence replay, and preserved human approval boundary.

### 2.3. Research Gap

Prior work separately demonstrates sensor-based rehabilitation assessment, agentic clinical reasoning, and automated scientific loops. The missing systems layer is a reproducible contract between hypothesis generation and embodied evidence acquisition. A useful framework must answer: What exactly is being proposed? Which sensor channels and features does it require? What evidence class does the response belong to? Who can veto or approve it? Is repeated execution safe? Can an independent process reconstruct the posterior from the record? EARL is designed around these questions.

The distinction from medical-agent systems is operational rather than a claim that multiple agents or traceability are new. DeepRare integrates phenotype, genomic, and retrieved medical evidence to rank diagnostic hypotheses with traceable reasoning [6]. RareAgents coordinates an LLM-based multidisciplinary team, memory, and medical tools for diagnosis and treatment decision support [7]. EARL instead selects a typed next evidence request and subjects any adapter execution to an independently enforced deterministic envelope. Its evaluated critics are fixed scoring functions, not clinical specialists or LLMs; its output is a replayable sequence of disclosures and posterior states, not a diagnosis or treatment plan. Diagnostic accuracy results from those systems are consequently not comparable with EARL’s four-cohort surrogate task. The present contribution is the executable selection–authorization–replay contract and its bounded evaluation.

## 3. Materials and Methods

### 3.1. Study Design and Evidence Boundary

This was a retrospective computational methods study. The empirical component used a public, de-identified gait dataset. The sequential process was a replay experiment: each probe revealed a predeclared bundle of features derived from one held-out subject’s existing recording. Robustness perturbations and active-response experiments were simulated and marked as such. Figure 1 shows the authority boundary.

Every boundary object is an immutable, strict schema. Hypotheses contain a label, description, and prior. A probe declares its family, required sensor channels, feature names, estimated cost, rationale, risk class, and optional typed actuation. Observations carry a timestamp, source reference, evidence origin, and—for simulated values only—a seed. Unknown fields, non-finite numbers, inconsistent probability distributions, duplicate identifiers, and missing provenance fail validation. Agent outputs are constrained to score cards with a score in [−1,1], a rationale, and an optional veto.

Three evidence origins are used throughout: observed for source measurements or their replayed values, derived for deterministic features, posterior states, statistics, and safety decisions, and simulated for robustness perturbations or digital-twin responses. These origins appear in contracts, ledger events, figures, captions, and release artifacts.

### 3.2. Dataset, Acquisition, and Cohorts

GaitNDD version 1.0.0 (DOI: 10.13026/C27G6C) was acquired from the authoritative PhysioNet repository [13]. The dataset contains 64 subjects: 13 with ALS, 20 with Huntington’s disease, 15 with Parkinson’s disease, and 16 controls. Each selected record comprises a metadata header, left- and right-foot force-derived series, and a 13-column stride-time file. The acquisition script downloads to temporary paths, verifies every file against the publisher’s SHA-256 manifest, and atomically renames successful downloads. The repository excludes raw data but retains the source version, DOI, license, selected-file list, sizes, and checksums in a machine-readable manifest.

The analysis unit was the subject. Subject identifiers were unique, and all files belonging to a subject remained in the same outer fold. Demographic variables were parsed for integrity checks but were not used as predictors. All learned means, variances, feature scales, and simulator ranges were fitted only on training subjects.

### 3.3. Feature Extraction and Probe Construction

The stride table contains time; left and right stride, swing, and stance durations; swing and stance percentages; and double-support duration and percentage. Twenty-five finite subject-level features were derived without using cohort-external data. They were grouped into six evidence families and exposed through eight probes (Table 1). Repeated probes within stance–swing and variability families permit the diversity critic to distinguish a new family from a second bundle in an already used family.

For a sequence $z_{1},\ldots ,z_{n}$, the coefficient of variation was the sample standard deviation divided by the absolute mean. Bilateral asymmetry was the mean absolute left–right difference divided by their pointwise mean magnitude. Long-range features included lag-one correlation and a compact detrended-fluctuation-analysis slope over feasible scales in {4,8,16,32,64}. The outlier fraction used a median-absolute-deviation robust *z*-score threshold of 4. Records with fewer than eight rows, a non-13-column shape, or non-finite values were rejected.

### 3.4. Probabilistic Evidence Model

The transparent reference model represents each class y∈Y and evidence family *f* by a diagonal Gaussian estimated inside the training fold. For features $x_{f}$ selected by a probe,

(1) $$p\left(x_{f}|y\right)=\prod_{j\in f}N\left(x_{j};\mu_{yfj},\sigma_{yfj}^{2}\right).$$

Class-specific variances were floored by the maximum of $10^{-3}$ times the global training variance and $10^{-8}$. Starting from a uniform prior, posterior updates were performed in log space:

(2) $$p_{t}\left(y\right)=\frac{p_{t-1}\left(y\right)p\left(x_{f_{t}}|y\right)}{\sum_{y^{\prime }\in Y}p_{t-1}\left(y^{\prime }\right)p\left(x_{f_{t}}|y^{\prime }\right)}.$$

Uncertainty was Shannon entropy in bits, $H\left(p_{t}\right)=-\sum_{y}p_{t}\left(y\right)\log_{2}p_{t}\left(y\right)$. The expected information gain of candidate probe *a* was

(3) $$\mathrm{EIG}\left(a\right)=H\left(p_{t}\right)-E_{x_{a}∼p\left(x_{a}|p_{t}\right)}\left[H\left(p_{t+1}\right)\right].$$

The expectation was approximated deterministically with 2d class-conditional sigma points for a *d*-feature probe, displaced by $\sqrt{d}$ standard deviations along each axis. EIG was clipped to $\left[0,H\left(p_{t}\right)\right]$. This model favors auditability over representational capacity; it is not proposed as a clinical classifier.

### 3.5. Hypothesis Council and Probe Planner

Five stateless critics independently produce validated score cards for every candidate (Table 2). Independence here means that critics share no mutable state and cannot read one another’s output; it does not imply statistical independence. A malformed or missing score card aborts planning.

Non-vetoed scores $s_{i}\left(a\right)$ are aggregated with explicit positive weights $w_{i}$:

(4) $$S\left(a\right)=\frac{\sum_{i}w_{i}s_{i}\left(a\right)}{\sum_{i}w_{i}}.$$

The complete EARL configuration used equal weights. Deterministic tie-breaking used aggregate score, EIG, acquisition cost, and probe identifier, in that order. The agent layer has proposal authority only.

This critic set is a reference design, not a task-independent optimum. Informativeness and falsification represent uncertainty reduction and separation of the leading alternatives; diversity discourages repeated families; feasibility represents declared resources and availability; and the safety critic screens risk permissions before the independent governor applies detailed constraints. Equal weights were fixed for the original benchmark and were not fitted to held-out labels. In the clean benchmark, all probes are passive and have identical unit cost, all required channels are available, and declared missingness is zero. Consequently, feasibility scores are identical across candidates at each decision and safety scores equal one; these roles cannot discriminate between clean candidates or establish a safety benefit there.

For another task, the protocol must first define admissible actions, feature families, a resource unit, and non-negotiable safety limits. Relevant scoring criteria and any unequal weights should then be specified in advance or selected using training-only inner validation against a declared multi-objective endpoint, frozen before independent evaluation, and assessed by ablation and sensitivity analysis. A safety veto remains binding regardless of weights, and the governor cannot be traded against information gain. This study does not estimate transferable weights for other rehabilitation tasks.

### 3.6. Safety Governor, Simulator, and Adapter Boundary

The deterministic safety governor revalidates a probe independently of council scoring and binds its decision to a SHA-256 hash of the complete safety envelope. The envelope covers cadence scaling, assistance level, and resistance level; it declares ranges, units, maximum duration, maximum step change, cumulative exposure, prerequisites, forbidden combinations, and risk classes that require explicit human approval. An emergency stop, missing prerequisite, unsupported actuation, invalid unit or range, excessive duration, rate violation, cumulative violation, or forbidden combination produces a rejection. Moderate and high-risk require approval. No generative output can override the result.

An approved probe and its matching safety decision are wrapped in a typed object before they reach an adapter. The replay adapter returns only features declared by the approved probe. The simulator adapter can instead apply a declared in-silico response model fitted on training subjects: active effects are sampled from predeclared sensitivity ranges, additive noise is bounded by training variance, and every value is clipped to at most 25% relative perturbation from baseline. Simulated observations require a seed and cannot be mislabeled as observed. The active-response simulator was implemented and tested as an architectural boundary; the primary benchmark used passive replay and never issued an actuation.

The frozen safety envelope permitted cadence ratios 0.90–1.10, assistance fractions 0–0.40, and resistance fractions 0–0.30 under clinician-supervision prerequisites, with additional duration and transition limits. These are software-test bounds, not a clinical protocol, device certification, or assertion of safe exposure.

### 3.7. Closed-Loop Execution and Evidence Ledger

Figure 2 summarizes the state machine. A run registers priors and candidates, ranks remaining probes, applies stopping rules, records the selected proposal, evaluates safety, executes an approved adapter call exactly once, records the observation, updates the posterior, and repeats. Terminal reasons are confidence, marginal information, maximum probes, cost, safety stop, or absence of a valid probe.

Adapter calls are protected by an idempotency key derived from the run identifier, step index, and canonical probe content. A repeated call with the same key returns the cached observation; the same key with different probe content raises an error. Ledger events use canonical JSON, timezone-aware timestamps, a parent event identifier, and a SHA-256 content identifier. Replay verifies each parent link and hash, then recomputes every posterior from recorded observations. A run is accepted for analysis only if the replayed posterior path equals the original path.

### 3.8. Evaluation Design

Repeated stratified four-fold cross-validation with five repeats produced 20 outer splits (Figure 3). In each split, the likelihood model, variance floors, perturbation scales, and randomized-feedback model were fitted only on training subjects. Each held-out subject was evaluated under 11 predeclared method–scenario conditions, yielding 3520 subject-condition rows. Every condition accepted exactly five unit-cost probes, so entropy trajectories had a common baseline and five post-probe states.

The five clean comparators were (i) fixed family order; (ii) a seeded random order; (iii) EIG, containing only the informativeness critic; (iv) complete EARL; and (v) a non-deployable oracle. The oracle had access to each held-out subject’s realized feature values and used exact dynamic programming over the remaining probes to maximize the final trapezoidal entropy-reduction area. It is therefore an upper reference, not a usable policy.

Three clean ablations removed the falsification critic, diversity critic, or simulator-informed informativeness critic. Three robustness conditions retained complete EARL while introducing Gaussian feature noise with a standard deviation 10% of the training-fold feature standard deviation; independent 20% feature missingness with at least one retained feature per probe; or a training-fold cohort-label permutation as a random-feedback negative control. Perturbed observations were labeled simulated and stored with their deterministic seeds.

### 3.9. Outcomes and Statistical Analysis

The primary endpoint was area under cumulative entropy reduction (AUC) across the five probes:

(5) $$A=\sum_{t=1}^{5}\frac{\left[H\left(p_{0}\right)-H\left(p_{t-1}\right)\right]+\left[H\left(p_{0}\right)-H\left(p_{t}\right)\right]}{2}.$$

Higher values indicate earlier and larger reduction in model uncertainty. Secondary endpoints were final macro accuracy, unscaled multiclass Brier score, top-label expected calibration error (ECE) over 10 equal-width bins, the fraction of subjects whose true-label posterior reached 0.8 at any step, and median probes to that threshold among subjects that reached it. Under the frozen aggregation, a subject reaches the threshold if at least one of its five repeated held-out runs reaches it. For each such subject, the first-hit probe counts are summarized by their median over reaching runs; the reported count is the median of those subject medians. Non-reaching runs do not enter the conditional count. Thus, threshold attainment is an any-repeat retrospective summary, not a single-run success probability. It uses the true cohort label only for evaluation and cannot serve as an operational stopping rule. These endpoints characterize the surrogate cohort-identification task and do not measure clinical diagnostic utility.

The five repeated observations for each subject and condition were aggregated within the subject before descriptive or inferential statistics. Mean AUC and paired AUC differences used percentile subject bootstrap intervals with 10,000 replicates. Each complete-EARL comparison used a two-sided paired sign-flip permutation test with 100,000 Monte Carlo replicates and the plus-one correction. Holm adjustment controlled the family-wise error rate across all ten original comparisons. Standardized paired effects divided the mean within-subject difference by its sample standard deviation. The original 11 conditions and frozen results were retained unchanged. Reviewer-requested analyses introduced during revision are explicitly post hoc, are stored separately, and are not additional confirmatory comparisons in that family.

### 3.10. Post Hoc Analyses Requested During Revision

The missing threshold endpoints were recovered from the original frozen rows using the original subject aggregation. Additional descriptive analyses count each probe’s inclusion among the five accepted selections and among the 320 run paths per policy (64 subjects times five repeats); these repeated paths are not independent participants. A clean-EARL critic audit reconstructs the original training folds and decision states and verifies its selected sequence against the frozen result. At each of the 1600 decisions it removes one critic while retaining the same posterior, remaining candidates, and tie-breaking rules. A changed first-ranked candidate measures local ranking sensitivity, not the downstream effect of rerunning the entire policy. Score ranges and veto counts distinguish inactive constraints from effective ranking criteria. Subject-bootstrap intervals for selected secondary metrics describe uncertainty conditional on the existing cross-validation predictions.

A sensitivity analysis removes the record-quality bundle from the candidate set of every clean policy and reruns the five policies on the original subjects, outer splits, fitted models, and seeds, so that no policy can select it at any step. Subject-aggregated entropy-reduction AUC, macro accuracy, Brier score, and expected calibration error are compared with the frozen original results, and paired within-subject AUC differences receive subject-bootstrap intervals. Fixed ordering never reached the sixth family within five probes and is reproduced unchanged, which verifies the rerun path. The rerun changes both the available evidence and the selection sequence; it is descriptive, post hoc, and cannot establish whether the removed features carry physiological or protocol information.

A separate resource scenario assigns illustrative costs to feature bundles and compares fixed ordering and complete EARL under a five-probe horizon with EARL using an operational confidence stop. The stop uses the maximum predicted class posterior, reaching 0.8, regardless of whether that class is correct; true labels are used only for evaluation. These are explicitly post hoc resource assumptions, not measured examination times, patient burden, or a cost-optimal policy. A second, fully simulated example exercises the complete loop when the candidate ranked first by complete EARL, and also by a diagnostic EIG-only ranking, violates the governor’s envelope. Its rejection, valid alternative, simulated observation, posterior update, and replay verification are recorded together. Neither scenario changes the original benchmark, and neither supplies new participant or physical-robot evidence. Configuration, detailed results, and reproduction commands appear in the Supplementary Materials.

### 3.11. Implementation and Reproducibility

The reference implementation uses Python 3.12, NumPy 2.5.0, SciPy 1.18.0, scikit-learn 1.9.0, Pydantic 2.13.4, and Matplotlib 3.11.0. Configuration parsing is strict and rejects unknown fields. The benchmark digest includes the complete evaluation configuration, safety-envelope content, and source bytes of the evaluation, agent, loop, model, planner, safety, and statistics modules. Checkpoint rows are atomically replaced and can be resumed only when their digest matches. Figure values and manuscript macros are generated from frozen JSON/CSV artifacts rather than transcribed manually.

The released workflow provides commands for checksum-verified data acquisition, tests, benchmark replay, figure generation, manuscript compilation, claim audit, and release packaging. Raw source data remain outside version control; the manifest and DOI permit independent reacquisition.

## 4. Results

### 4.1. Run Integrity and Primary Endpoint

All 3520 configured rows were present, all ledger chains verified, and all test subjects were absent from their training-subject lists. Complete EARL achieved a mean entropy-reduction AUC of 7.689 (95% CI 7.535–7.840). It exceeded fixed ordering by 0.459 (95% CI 0.255–0.660; Holm-adjusted p=0.00010) and random ordering by 0.763 (95% CI 0.609–0.919; adjusted p=0.00010). It was lower than EIG by −0.321 (95% CI −0.426–−0.217; adjusted p=0.00010) and lower than the oracle by −1.052 (95% CI −1.180–−0.930; adjusted p=0.00010). The negative differences indicate that the comparator had higher AUC. Table 3 summarizes the clean-condition outcomes for all five policies.

Although EIG reduced entropy faster, complete EARL had descriptively higher macro accuracy (55.8% versus 49.5%), lower Brier score (0.781 versus 0.813), and lower ECE (0.364 versus 0.398). These secondary estimates do not establish a statistically confirmed advantage. The primary comparison quantifies the entropy efficiency relinquished when other selection criteria are added to EIG: the policies are evaluated on an endpoint closely aligned with EIG’s one-step objective. Figure 4 visualizes the cumulative entropy reduction and the corresponding subject-aggregated AUC.

### 4.2. Threshold Attainment and Selection Frequencies

Table 4 supplies the predeclared threshold endpoints omitted from the original results. EARL reached a true-label posterior of 0.8 in at least one repeat for 49 of 64 subjects (76.56%), with a conditional median of two probes; EIG reached it for 52 subjects (81.25%), with a median of one probe. Run counts are also shown to expose the difference between any-repeat attainment and a single-run outcome. The randomized-feedback control reached the subject endpoint for 55 of 64 subjects (85.94%) but only 106 of 320 runs (33.13%). Repeated opportunities to cross the true-label threshold can therefore yield a high subject fraction even under a poor predictive model; the endpoint is neither sustained correctness nor clinical success.

Within the two-bundle families, EARL selected phase percentages in 176 and double support in 160 of its 1600 selections (11.00% and 10.00%), whereas stride–swing variability accounted for 306 selections (19.13%) and double-support variability for only 16 (1.00%), in 11 of 64 subjects. EIG and the oracle used double-support variability more often (5.44% and 7.38%), and fixed ordering never reached that bundle, long-range dynamics, or record quality within five probes. Record quality accounted for 186 of EARL’s 1600 selections (11.63%) and occurred in 186 of 320 run paths (58.13%), involving 52 of 64 subjects in at least one repeat. EIG selected it in 152 paths (47.50%), random ordering in 205 (64.06%), the oracle in 169 (52.81%), and fixed ordering in none. Table 5 reports the complete probe-bundle distribution. These frequencies establish use, not the absence of recording-protocol bias. Complete condition-level counts and denominators accompany the source archive.

### 4.3. Sensitivity to Record-Quality Exclusion

Table 6 compares each clean policy with all eight bundles and with the record-quality bundle excluded from selection. Excluding it lowered mean AUC by 0.035 for EARL (95% CI −0.017–0.092), by 0.041 for EIG (−0.015–0.101), and by 0.035 for the oracle (0.011–0.068); fixed ordering was unchanged because it never reached the sixth family. Macro accuracy did not decrease for any policy: EARL rose from 55.8% to 57.1% and EIG from 49.5% to 53.9%, while Brier scores fell (EARL 0.781 to 0.767; EIG 0.813 to 0.778) and ECE changed by at most 0.04. The EARL-minus-EIG AUC difference without record quality was −0.316 (95% CI −0.420–−0.209; adjusted p=0.00004), against −0.321 originally. EARL reached the true-label threshold for 73.44% of subjects (originally 76.56%), and 134 of its 320 family paths were unchanged. The primary comparisons therefore do not depend on the record-quality bundle, but the small AUC reductions confirm that it carried usable information whose origin remains unresolved.

### 4.4. Ablation Results

Removing the simulator-informed informativeness critic reduced AUC by 0.475 in the EARL-minus-ablation contrast (95% CI 0.288–0.663; adjusted p=0.00010). Removing the diversity critic produced a negligible primary-endpoint difference of −0.004 (95% CI −0.023–0.014; adjusted p=0.66317), although the complete policy’s macro accuracy was 55.8% compared with 48.3% for the ablation. Removing the falsification critic increased AUC: the complete-minus-ablation difference was −0.316 (95% CI −0.423–−0.210; adjusted p=0.00010). The no-falsification policy nevertheless had a higher Brier score (0.810 versus 0.781). These results reject a simple claim that every added critic improves every metric.

### 4.5. Post Hoc Critic Influence and Subject Uncertainty

The critic audit reproduced all 320 clean-EARL paths before examining 1600 decision states. Removing informativeness, falsification, or diversity changed the first-ranked candidate in 771, 525, or 232 states, respectively (Table 7). Removing feasibility or safety changed no selections. Feasibility scores ranged from 0 to 0.8 across steps but had zero range across candidates at any given state; safety scores were uniformly 1. All five critics emitted zero vetoes over 9600 candidate score cards per critic. Thus, only three critics affected clean-benchmark rankings. These one-step sensitivities hold the actual state fixed and differ from the full-path ablations above.

Post hoc cohort-stratified subject bootstrap intervals for macro accuracy were 45.08–66.74% for EARL and 39.01–59.78% for EIG. The wide intervals underscore the limited precision of this 64-subject comparison. Omitting one subject at a time from the existing paired AUC aggregates left the EARL-minus-EIG difference between −0.341 and −0.302. This is a descriptive influence analysis without refitting, not external validation. Supplementary Section S13 reports the corresponding Brier intervals and resampling details.

### 4.6. Robustness and Random-Feedback Control

Feature missingness reduced complete-EARL AUC from 7.689 to 7.343, a paired difference of 0.346 (95% CI 0.237–0.461; adjusted p=0.00010). Added feature noise reduced AUC to 7.284, a difference of 0.405 (95% CI 0.254–0.553; adjusted p=0.00010), and macro accuracy fell to 45.8%. Figure 5 summarizes the clean-condition secondary metrics and the simulated robustness conditions.

The randomized-training-label control produced AUC 7.022 and macro accuracy 25.1%, approximately the 25% level expected for four balanced evaluation classes. This control shows that the pipeline can still reduce the entropy of a mis-specified model while losing meaningful correspondence with the held-out labels. Entropy reduction must therefore be interpreted together with predictive and calibration endpoints.

### 4.7. Safety Challenge Conformance

All 10 of 10 predeclared safety cases returned their expected outcome. The valid cadence proposal was approved. Out-of-range value, excessive duration, unit mismatch, rate change, cumulative exposure, forbidden assistance–resistance combination, missing supervision prerequisite, and emergency-stop cases were rejected. Figure 6 shows expected versus observed outcomes.

### 4.8. Post Hoc Resource and End-to-End Safety Scenarios

Illustrative costs were one unit for stride timing, asymmetry, and record quality; two for stance–swing and variability; and three for long-range dynamics, per the accepted bundle. All arms used the same subjects, splits, and a non-binding 15-unit budget. Costs affect EARL’s feasibility score, so these are separate scenario results, not replacements for the original unit-cost outcomes. EARL’s predicted-posterior stop reduced mean probes from 5.000 to 1.722 and illustrative cost from 7.956 to 2.581 units, while macro accuracy decreased from 55.81% to 48.26% (Table 8). All 320 early-stop paths ended at the confidence threshold. Lower Brier and ECE values coexist with lower accuracy; this illustrates a resource–prediction trade-off and does not establish clinical savings or an optimal stopping policy.

In the constructed active-response example, complete EARL ranked the unsafe candidate first (score 0.736 versus 0.635); the same candidate also had higher estimated EIG (1.590 versus 0.536 bits). Its requested cadence ratio of 1.20 exceeded the illustrative 0.90–1.10 envelope and was rejected with value_out_of_range. The loop then selected the valid 1.04-ratio alternative, executed it through the simulator, recorded a simulated observation, and updated the posterior. The rejected proposal was never executed, and the eight-event ledger replayed exactly. Both candidates had equal illustrative cost and low-risk declarations; the governor independently enforced the numeric envelope. This single constructed case demonstrates a changed execution sequence under complete EARL, not the prevalence of unsafe choices or physical safety.

## 5. Discussion

### 5.1. Principal Findings

EARL provides a concrete architecture for adaptive evidence selection: a research loop can propose the next feature disclosure, estimate its value, execute only within a deterministic envelope, update uncertainty, and preserve enough evidence to replay the result. The original implementation completed 3520 sequential runs without subject leakage or ledger mismatch. The complete policy improved the primary endpoint over fixed and random ordering, while its failure to exceed pure EIG prevents an inflated claim of multi-agent superiority. Because all primary conditions consumed five unit-cost probes, that experiment demonstrates ordering-dependent information efficiency, not a reduction in measurements, examination time, or patient burden.

EIG greedily maximizes expected one-step entropy reduction, whereas the primary endpoint integrates realized entropy reduction across five steps. These are closely aligned, although greedy EIG does not mathematically optimize the complete realized trajectory. Its observed advantage is therefore consistent with the chosen criterion. EARL gives up some entropy efficiency to additional scoring preferences; in the clean benchmark only informativeness, falsification, and diversity discriminate between candidates. Feasibility and safety are architectural constraints whose roles require other scenarios. The descriptively better accuracy and Brier/ECE values do not establish clinical utility or a reliable predictive advantage in a new cohort. The appropriate conclusion is a measured trade-off under this model and dataset.

The ablations further show that agent roles require empirical justification. The simulator-informed critic contributed substantially to entropy efficiency. Diversity had little effect on AUC but coincided with better macro accuracy, which may reflect reduced repeated-family evidence. Falsification lowered primary AUC relative to its ablation but improved Brier score. A future system should therefore treat council composition and weights as registered, testable design choices rather than intuitive necessities.

### 5.2. Implications for Robotic Rehabilitation Research

The same contract could represent a new passive sensor request or an active, bounded robotic experiment, but that capability is distinct from the present retrospective feature disclosure. Table 9 maps every current probe to a possible future measurement or quality check. These mappings are design examples, not validated acquisition protocols. Several bundles could be computed from the same future recording; counting each as a separate measurement or adding their costs would then overstate resource use. A prospective system must model shared acquisition, recording length, available channels, and the task actually performed.

In prospective research, resource use could mean sensor occupancy, acquisition duration, computation, or a protocol-defined burden measure. None of these was measured by the original unit-cost benchmark. The post hoc scenario demonstrates how explicit costs and early stopping can alter software behavior, but translating its resource units into time or burden requires independent measurement and validation of the stopping rule. A high posterior can be confidently wrong and cannot authorize a clinical decision.

In a clinical research setting, a candidate might request synchronized force, inertial, electromyographic, or interaction-torque evidence under a clinician-defined task. The proposal layer could simulate multiple responses and score the value of competing probes. Crucially, the generative or multi-agent layer would still be unable to issue a device command: protocol validation, current state, prerequisites, explicit approval, idempotency, and emergency-stop state would be checked by deterministic code at execution time.

This arrangement does not remove the need for clinician judgment. Instead, it makes the handoff inspectable. A reviewer can see which hypotheses were active, why each critic scored a probe, whether any critic vetoed it, which envelope version governed the decision, what the adapter returned, and how that observation changed the posterior. Rejected probes remain evidence rather than disappearing from the record.

The path to prospective use is staged in Figure 7. The current evidence supports only retrospective replay and in-silico boundaries. Hardware shadow mode would next test vendor adapters, timing, disconnects, stale state, and fault injection without actuation. A supervised feasibility study would require a registered protocol, an independently reviewed envelope, explicit approval at each step, and prospective endpoints. Clinical utility and patient safety would require separate, adequately powered evaluation and monitoring.

### 5.3. Limitations

First, GaitNDD is a small historical dataset: the independent observational units are 64 people, not 3520 independent cases. Each outer split uses 48 training and 16 test subjects, and the four cohorts contain only 13–20 people each. Repeated folds assess sensitivity to partitioning; they do not increase the number of participants or provide external replication. Training-fold isolation limits leakage, but cannot remove cohort composition, acquisition-protocol confounding, or sampling uncertainty. Bootstrap intervals are conditional on the fitted cross-validation predictions and do not refit the full learning pipeline or capture between-institution variation; overlapping training sets also limit independence assumptions. The results are preliminary computational comparisons, not robust estimates of clinical performance. The four-way cohort task is not a differential diagnosis and cannot represent the breadth of rare or unexplained presentations.

Second, sequential selection was emulated by revealing feature bundles from one existing recording. It does not measure within-person repeatability, sensor repositioning, participant fatigue, adaptive task effects, or closed-loop latency. The record-quality bundle contains recording duration and stride count as well as missingness and outlier summaries. These can encode acquisition conventions or disease-associated movement patterns rather than purely technical quality. Its use for cohort prediction can therefore create a shortcut that fails outside GaitNDD. Selection frequency is descriptive and cannot identify whether its predictive information is physiological or protocol-driven. The exclusion analysis shows that the primary comparisons and the secondary accuracy and calibration metrics do not depend on this bundle, but it does not show that the remaining bundles are free of protocol information, and the small AUC reductions confirm that the removed features were informative for this dataset. Independent acquisition-protocol validation, including exclusion or restriction of quality variables to availability checks, is needed before transfer.

Third, the model assumes diagonal class-conditional Gaussians and conditional independence across sequential updates. This transparent baseline may be miscalibrated and cannot capture complex temporal structure. ECE estimates from 64 subject aggregates are themselves uncertain; we report them descriptively. Fourth, the deterministic council demonstrates the contract and evaluation method but not language-model reasoning quality. Replacing a critic with a generative agent would require output validation, model/version logging, adversarial testing, timeout handling, and repeated stochastic evaluation.

Fifth, the active-response simulator uses declared sensitivity ranges rather than causal estimates. It is a bounded scenario generator, not a digital patient and not evidence of treatment response. Sixth, the safety suite verifies code paths against one illustrative envelope. It does not address hardware faults, biomechanical hazards, cybersecurity, human factors, regulatory requirements, or prospective adverse events. Finally, the study uses one dataset and one primary acquisition objective. Independent datasets, modalities, institutions, and prospective protocols are needed before considering clinical translation.

## 6. Conclusions

EARL is a typed, replayable framework for adaptive sequential selection of evidence from pre-recorded gait data. The primary experiment establishes ordering-dependent information-selection behavior under a fixed five-probe budget; it does not demonstrate acquisition of new sensor measurements. In this small retrospective cohort, EARL reduced uncertainty earlier than fixed and random ordering, while pure EIG achieved the higher entropy-based primary endpoint. Secondary accuracy and calibration differences remain descriptive. Separate post hoc resource and safety scenarios illustrate software behavior under declared assumptions, rather than measured clinical savings or robot performance. The architecture preserves deterministic execution authority and explicit provenance, but prospective sensing, clinical diagnostic performance, therapeutic benefit, physical-robot safety, and patient efficacy all remain unestablished.

## Figures and Tables

**Figure 1:**
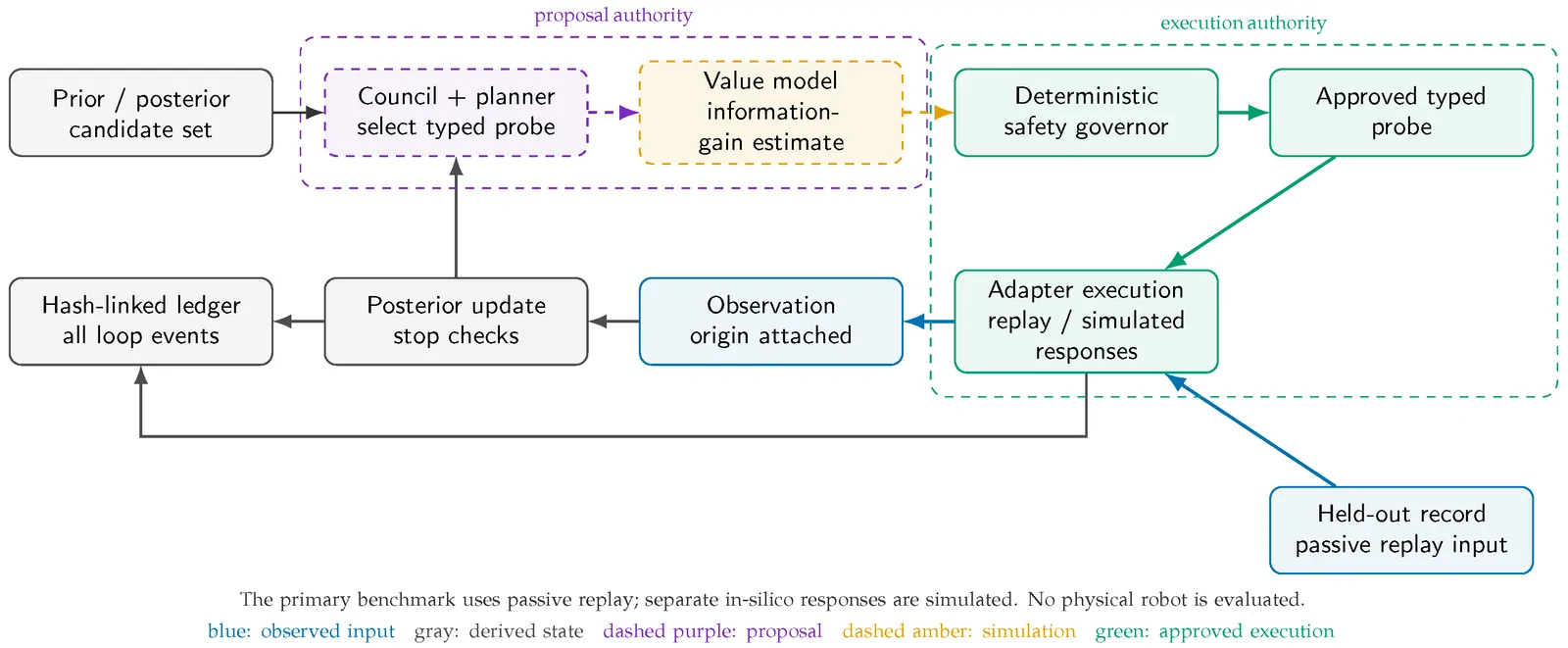
EARL architecture and authority boundary. The evaluated data path discloses features from existing gait recordings; the separate simulator returns explicitly simulated responses. Critic scores and proposals do not confer execution authority. The deterministic governor must approve an adapter call. A physical sensing adapter would require new validation and is not part of the evaluated deployment.

**Figure 2:**
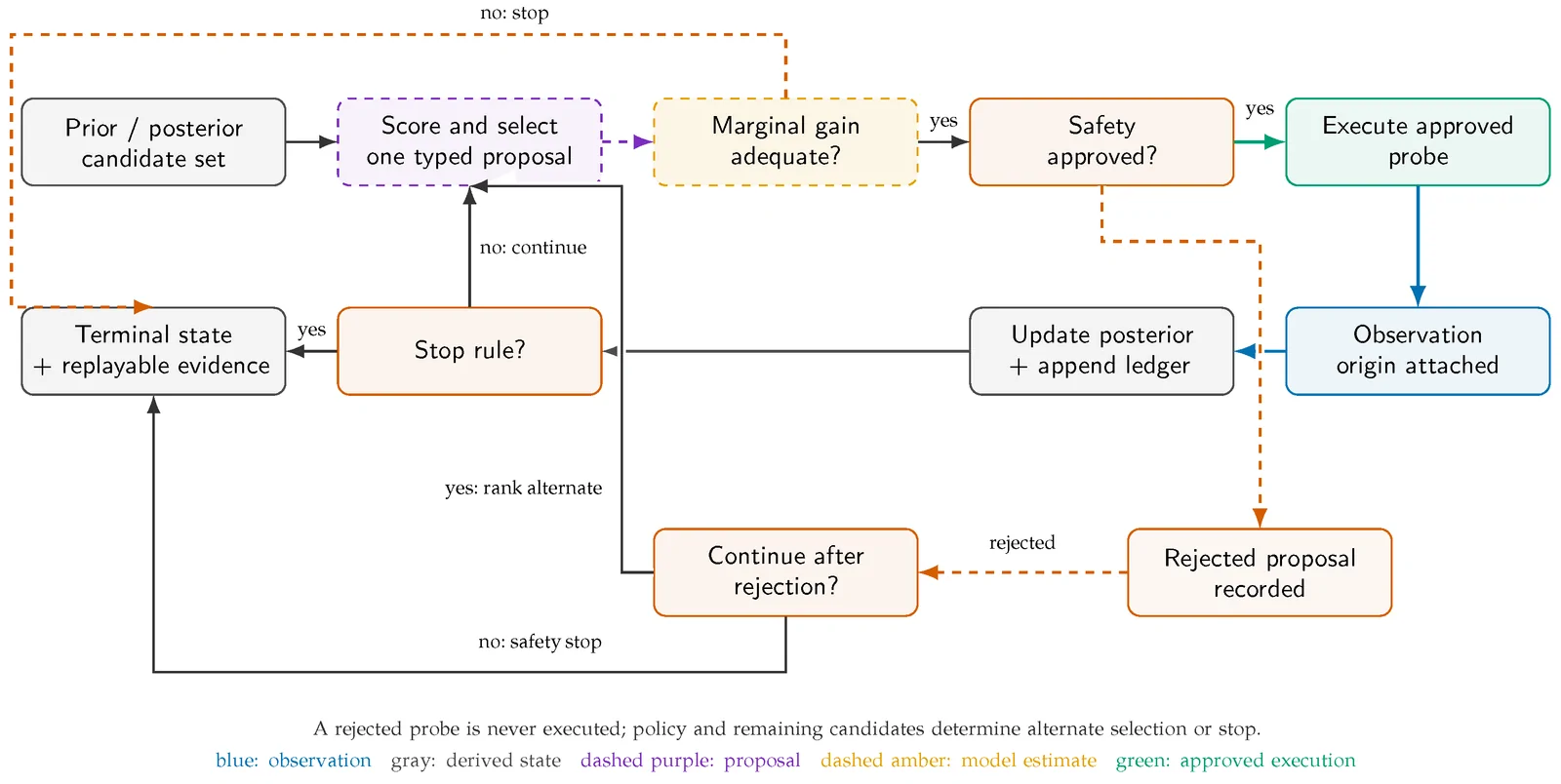
Closed-loop execution. Stopping checks precede candidate selection; the governor separates a proposal from approved execution. A rejected candidate is recorded and removed before replanning when continuation is permitted. Approved observations update the posterior. Evidence origins distinguish retrospective replay from simulated responses, and the complete event chain supports replay verification.

**Figure 3:**
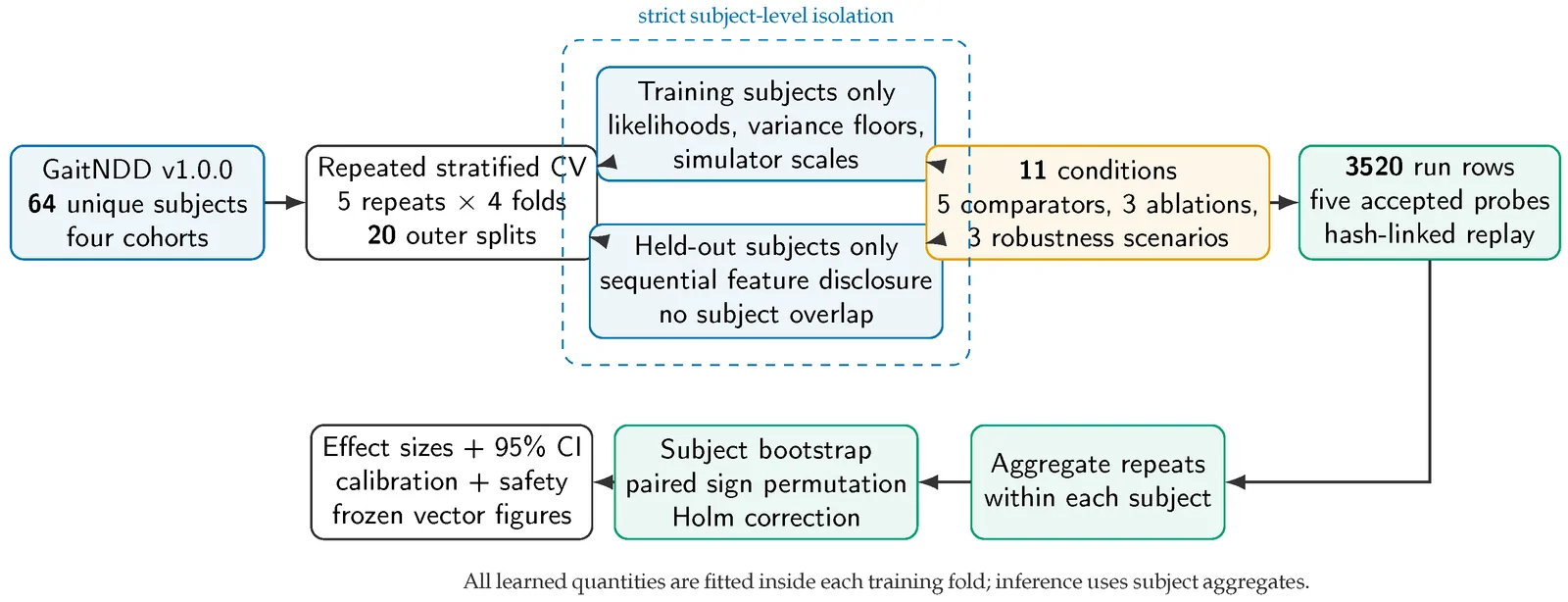
Leakage-controlled evaluation and subject-clustered inference. All learned quantities originate within a training fold; all statistical inference uses subject aggregates after repeated runs are combined.

**Figure 4:**
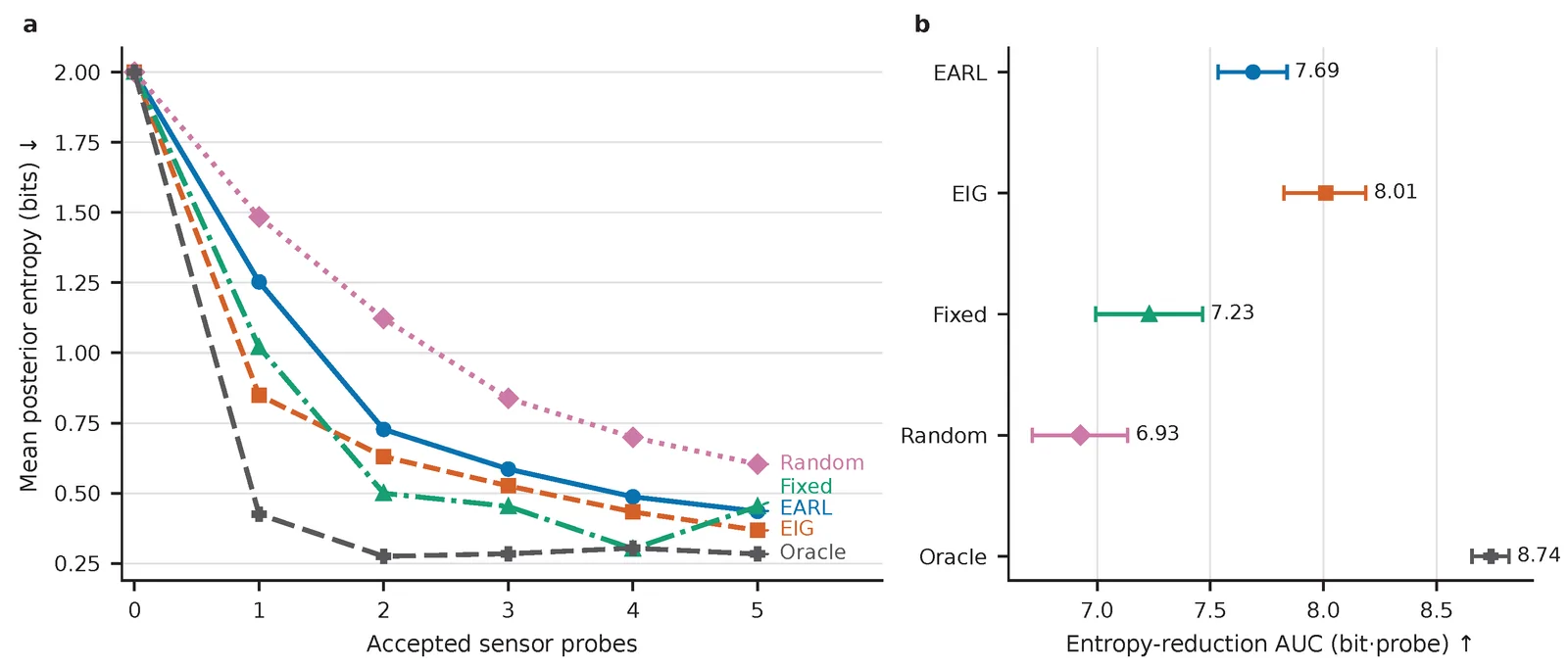
Primary endpoint. (**a**) Mean cumulative entropy reduction across five accepted probes; colors and line styles distinguish the five policies. (**b**) Subject-aggregated AUC point estimates with horizontal 95% bootstrap confidence intervals; the rightward direction indicates higher AUC and therefore better performance on this endpoint. Numeric content is generated from the frozen benchmark summary.

**Figure 5:**
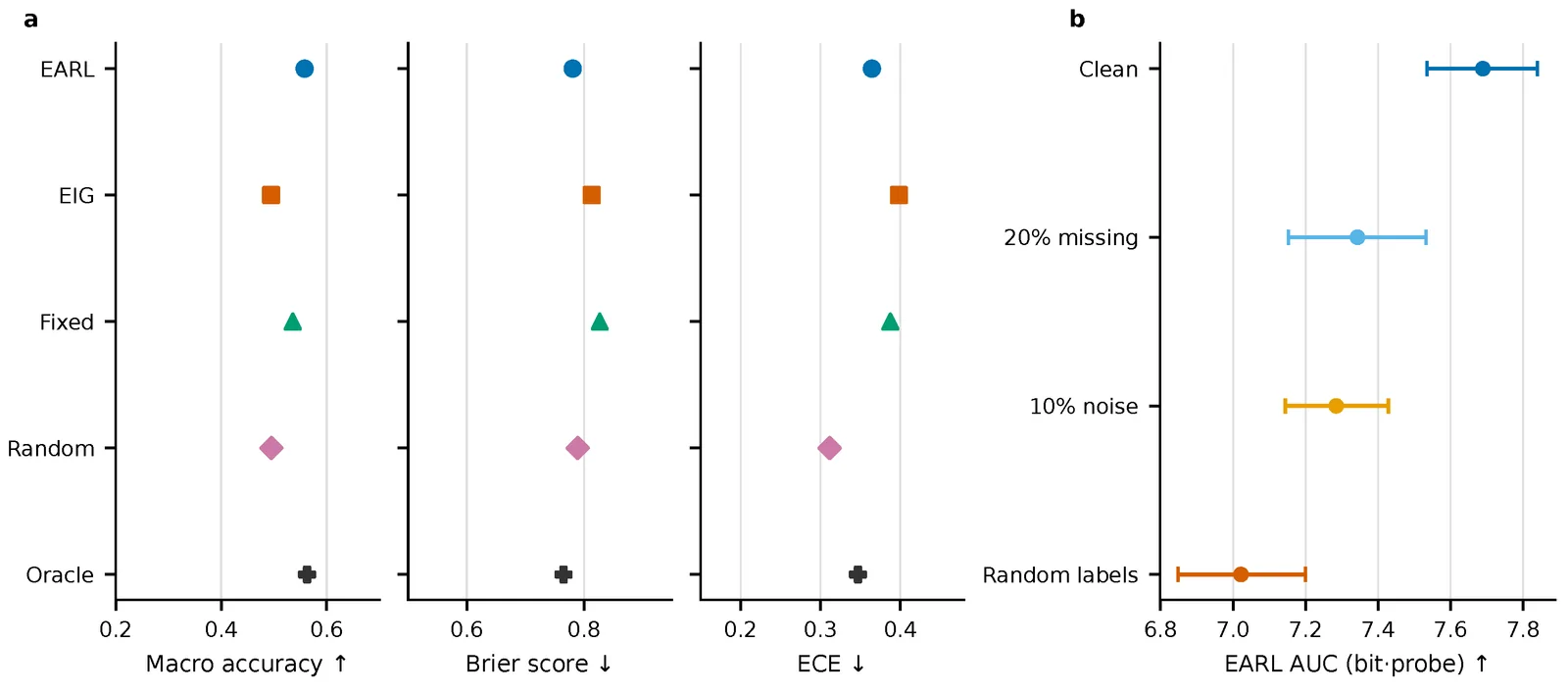
Secondary clean-condition metrics and robustness AUC. (**a**) Subject-aggregated macro accuracy, Brier score, and ECE for the five policies; marker colors and shapes identify the policies. Higher is better for accuracy, whereas lower is better for Brier score and ECE. (**b**) Complete-EARL AUC under the clean, 20% missingness, 10% noise, and randomized-training-label conditions; points show estimates and horizontal lines show 95% subject-bootstrap confidence intervals. Colors distinguish conditions, rightward values indicate higher AUC, and all perturbed conditions are simulated.

**Figure 6:**
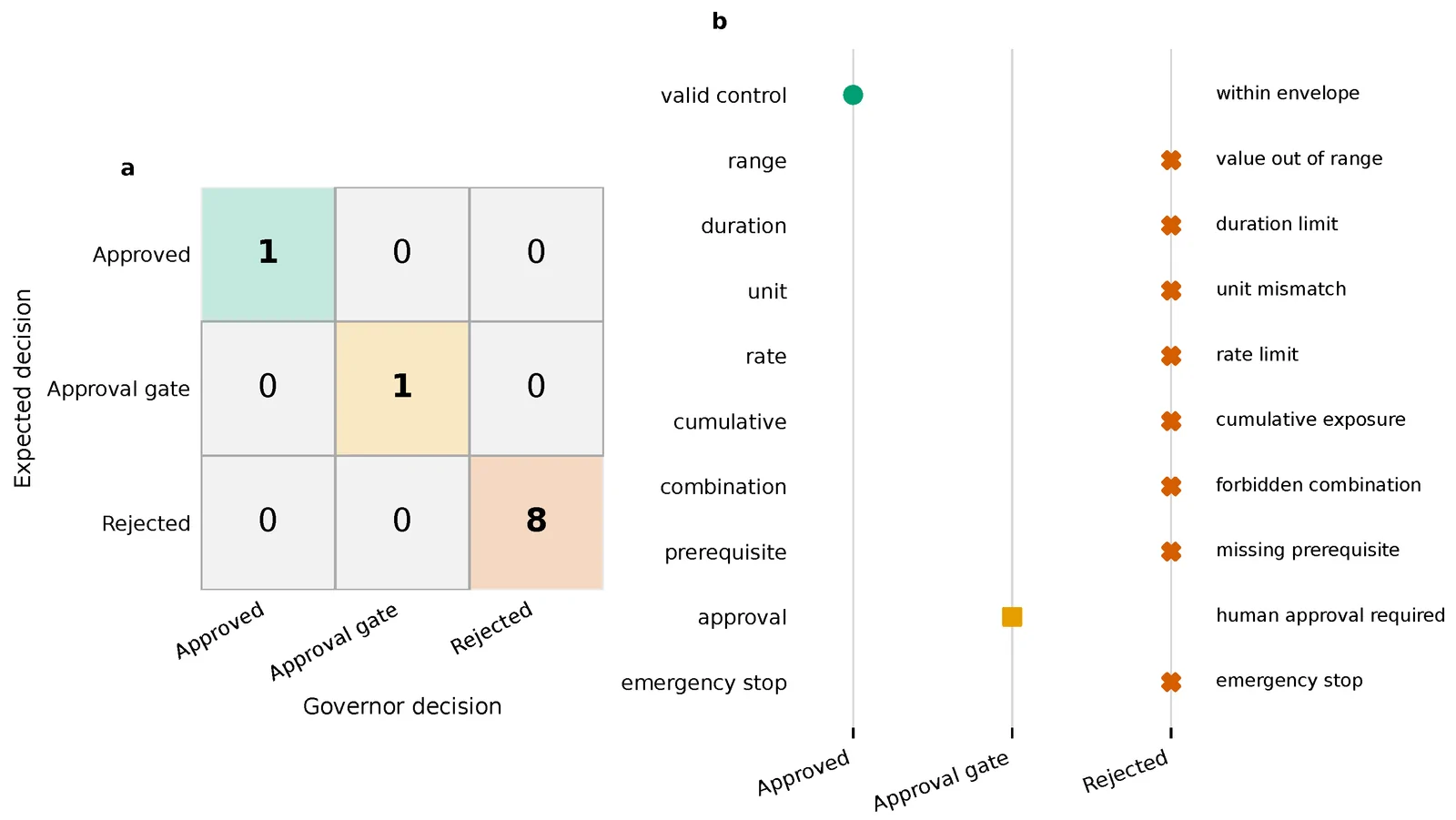
Deterministic safety-governor challenge suite. (**a**) Confusion matrix comparing expected and observed governor decisions; colored diagonal cells contain the counts of matching outcomes, and gray off-diagonal cells show mismatches. (**b**) Outcome of each challenged constraint; marker position gives the governor decision, marker color distinguishes approved (green), approval-gated (yellow), and rejected (orange) outcomes, and the adjacent label states the governing condition. A 100.0% software-test pass rate does not establish device or patient safety.

**Figure 7:**
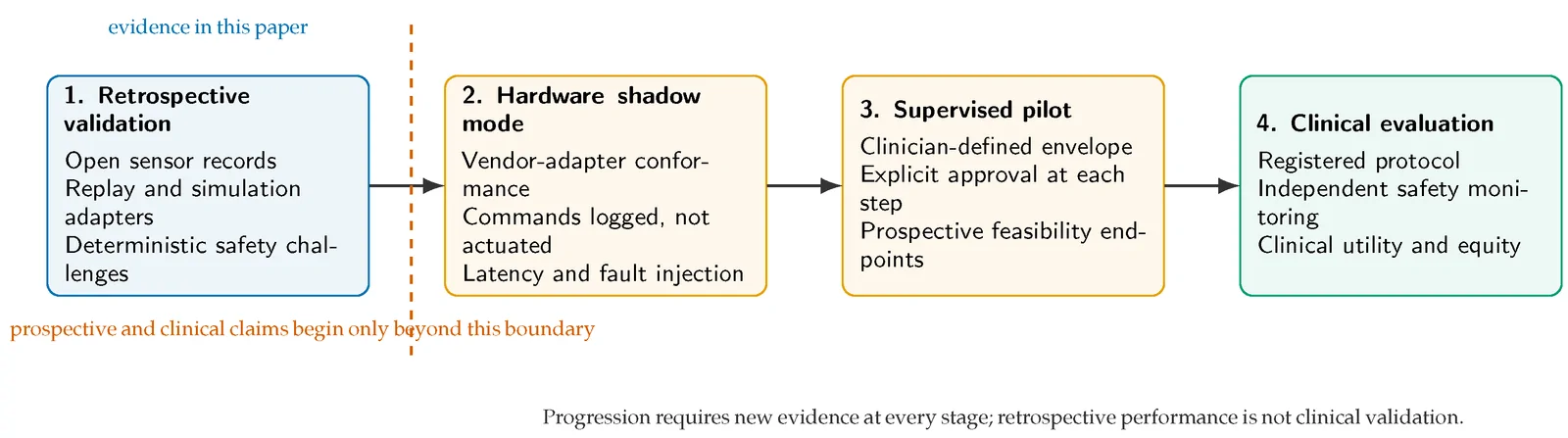
Evidence-gated deployment roadmap. Only the retrospective stage is evaluated here. Every later stage requires new prospective evidence; no performance result in this article crosses the dashed clinical-claims boundary.

**Table 1:** Predeclared feature families and sequential probe bundles. All quantities are derived from retrospective bilateral force-sensor gait records.

Family	Probe Bundle	Derived Features
Stride timing	Stride means	Left, right, and bilateral mean stride time
Asymmetry	Bilateral asymmetry	Normalized stride, swing, and stance asymmetry
Stance–swing	Phase percentages	Left/right mean swing and stance percentages
Stance–swing	Double support	Mean double-support duration and percentage
Variability	Stride–swing variability	Coefficients of variation for left/right stride and left/right swing
Variability	Double-support variability	Double-support coefficient of variation
Long range	Long-range dynamics	Left/right lag-one correlations and detrended-fluctuation slopes
Signal quality	Record quality	Stride count, duration, missing fraction, and robust stride-outlier fraction

**Table 2:** Council roles in the complete EARL policy. A veto removes a candidate before aggregation.

Critic	Score	Veto Condition
Informativeness	EIG normalized by current entropy	None
Falsification	Hyperbolic tangent of standardized separation between the two leading hypotheses	None
Diversity	1−0.75k, clipped to [−1,1], where *k* is prior use count of the family	None
Feasibility	One minus cost fraction minus twice declared missingness	Missing channel or cost above remaining budget
Safety	Ordinal score from passive to high risk	Emergency stop, prohibited risk, or unapproved high risk

**Table 3:** Clean-condition outcomes. AUC is the primary endpoint (higher is better); Brier score and ECE are lower when better calibrated. Intervals are 95% subject-bootstrap intervals.

Policy	AUC (95% CI)	Macro Accuracy (%)	Brier	ECE
EARL	7.689 (7.535–7.840)	55.8	0.781	0.364
EIG	8.010 (7.826–8.186)	49.5	0.813	0.398
Fixed	7.230 (6.994–7.467)	53.6	0.827	0.387
Random	6.926 (6.713–7.134)	49.5	0.789	0.312
Oracle	8.741 (8.657–8.820)	56.3	0.765	0.347

**Table 4:** Predeclared threshold endpoints for clean policies. Subject attainment means at least one reaching repeat. The median is taken over reaching subjects after their first-hit counts are summarized across reaching repeats; its denominator is the Subjects column. All policies still executed five probes.

Policy	Subjects	Subjects (%)	Median Probes	Reaching Runs
EARL	49/64	76.56	2	186/320
EIG	52/64	81.25	1	196/320
Fixed	42/64	65.63	2	178/320
Random	50/64	78.13	2	180/320
Oracle	44/64	68.75	1	190/320

**Table 5:** Distribution of accepted probe-bundle selections in clean policies (% of 1600 selections per policy) for all eight bundles. Bundle identities were recovered by deterministic replay of every frozen row with its original fold, seed, and policy; each replay reproduced the frozen family sequence and posterior path, and bundle counts sum to the frozen family counts. Each column sums to 100% apart from rounding.

Family	Probe Bundle	EARL	EIG	Fixed	Random	Oracle
Stride timing	Stride means	17.88	12.31	20.00	12.44	11.44
Asymmetry	Bilateral asymmetry	18.88	18.44	20.00	12.75	15.88
Stance–swing	Phase percentages	11.00	11.44	20.00	12.31	12.81
Stance–swing	Double support	10.00	12.38	20.00	12.44	13.19
Variability	Stride–swing variability	19.13	19.69	20.00	12.56	15.75
Variability	Double-support variability	1.00	5.44	0.00	12.50	7.38
Long range	Long-range dynamics	10.50	10.81	0.00	12.19	13.00
Signal quality	Record quality	11.63	9.50	0.00	12.81	10.56

**Table 6:** Post hoc sensitivity analysis excluding the record-quality bundle from selection. Original rows are the frozen results; exclusion rows rerun each clean policy with seven candidates on the same subjects, splits, models, and seeds. Metrics use the 64 subject-averaged posteriors; AUC is the mean entropy-reduction area with a subject-bootstrap 95% CI.

Policy	Candidate Set	AUC (95% CI)	Accuracy (%)	Brier	ECE
EARL	All eight bundles	7.689 (7.535–7.840)	55.8	0.781	0.364
	Without record quality	7.654 (7.485–7.818)	57.1	0.767	0.361
EIG	All eight bundles	8.010 (7.826–8.186)	49.5	0.813	0.398
	Without record quality	7.969 (7.785–8.152)	53.9	0.778	0.366
Fixed	All eight bundles	7.230 (6.994–7.467)	53.6	0.827	0.387
	Without record quality	7.230 (6.996–7.470)	53.6	0.827	0.387
Random	All eight bundles	6.926 (6.713–7.134)	49.5	0.789	0.312
	Without record quality	6.892 (6.655–7.127)	53.9	0.740	0.274
Oracle	All eight bundles	8.741 (8.657–8.820)	56.3	0.765	0.347
	Without record quality	8.706 (8.611–8.795)	56.3	0.757	0.356

**Table 7:** Local influence of each critic in the clean EARL benchmark. Each removal is evaluated against the same original posterior, history, candidate set, and tie-breaking rule. Veto counts refer to original score cards.

Removed Critic	Changed Decisions/1600	Changed (%)	Vetoes/9600
Informativeness	771	48.19	0
Falsification	525	32.81	0
Diversity	232	14.50	0
Feasibility	0	0.00	0
Safety	0	0.00	0

**Table 8:** Post hoc heterogeneous-cost scenario. Probes and costs are run means over 320 paths per arm. Predictive metrics use the 64 subject-averaged posterior distributions. Costs are illustrative units, not measured acquisition time or patient burden.

Arm	Probes	Cost Units	Accuracy (%)	Brier	ECE
Fixed, five probes	5.000	8.000	53.57	0.827	0.387
EARL, five probes	5.000	7.956	55.81	0.774	0.362
EARL, confidence stop	1.722	2.581	48.26	0.747	0.303

**Table 9:** Possible future counterparts of the eight retrospective probes. No new measurements or robot tasks in this table were performed in the present study.

Current Feature Disclosure	Prospective Measurement or Check Requiring Validation
Stride means	Bilateral foot-contact timing over a specified walking segment
Bilateral asymmetry	Synchronized left–right stride, swing, and stance timing
Phase percentages	Foot-contact events sufficient to estimate relative swing and stance durations
Double support	Synchronized bilateral contacts sufficient to identify shared support intervals
Stride–swing variability	Repeated strides over a predefined observation window; estimate duration variability
Double-support variability	Repeated bilateral support intervals over a predefined window
Long-range dynamics	A sufficiently long continuous stride series; validate required length and stationarity
Record quality	Duration, completeness, and artifact checks for acceptance or reacquisition; avoid assuming these are disease-specific evidence

## Data Availability

The source data are publicly available from PhysioNet as the Gait in Neurodegenerative Disease Database version 1.0.0 at https://doi.org/10.13026/C27G6C. The submission archive contains the checksum manifest, MIT-licensed source code, configuration, frozen subject-level results, statistical outputs, figure sources, and reproduction instructions. Raw PhysioNet records are not redistributed and can be reacquired with the included checksum-verifying command. The private development repository will be made public or archived with a persistent identifier upon acceptance.

## Supplementary Materials

The following supporting information accompanies this article https://www.mdpi.com/article/10.3390/s26196120/s1, Supplementary Methods S1–S16, including feature definitions, contracts, agent equations, safety-envelope details, benchmark conditions, frozen checksums, post hoc revision analyses and scenarios, and complete reproduction commands. A source and reproducibility archive is included with the submission.

## Abbreviations

The following abbreviations are used in this manuscript:

ALS	Amyotrophic lateral sclerosis
AUC	Area under cumulative entropy reduction
CV	Cross-validation
EARL	Embodied evidence acquisition and reasoning loop
ECE	Expected calibration error
EIG	Expected information gain
FSR	Force-sensitive resistor
GaitNDD	Gait in Neurodegenerative Disease Database
SHA-256	Secure Hash Algorithm 256-bit
