# Supplementary material for: A Safety-Governed Architecture for Adaptive Sequential Evidence Selection from Pre-Recorded Gait Data
Source: Sensors (Basel). 2026 Sep 27;26(19):6120. doi: 10.3390/s26196120 (PMC13667541; doi:10.3390/s26196120)
Supplement: Supplementary file 1 [file sensors-26-06120-s001.zip › sensors-4520934-supplementary.pdf]

Supplementary Materials: A Safety-Governed Architecture for Adaptive Sequential Evidence Selection from Pre-Recorded Gait Data

Giulio Leone [1] and Daniela D'Auria [1,*]

S1. Scope and Evidence Classification

These Supplementary Materials specify the complete reproducibility boundary for the Embodied Evidence Acquisition and Reasoning Loop (EARL). The reference package distinguishes three evidence origins:

- **Observed**: values distributed by the PhysioNet source dataset or faithfully returned by the retrospective replay adapter.
- **Derived**: deterministic features, model states, statistics, safety decisions, and ledger identifiers computed from observed or simulated inputs.
- **Simulated**: robustness perturbations and bounded active-response scenarios produced by declared algorithms and seeds.

The benchmark contains observed replay and simulated robustness conditions. It contains no prospective participant measurements and no physical robot execution. Passing software tests does not establish clinical safety, diagnostic validity, or treatment efficacy.

S2. Reproducible Environment and Package Map

The frozen environment targets Python 3.12.12 and is resolved by `uv.lock`. The LaTeX source uses the official MDPI template archive dated 23 June 2026. Table S1 maps the principal release artifacts.

Table S1. Submission and reproducibility artifacts.

Path	Purpose
`manuscript.tex`	Main MDPI Sensors article
`supplementary-information.tex`	Standalone Supplementary Materials
`src/earl/`	Typed framework, adapters, simulator, safety governor, evaluation, and reporting
`config/benchmark.json`	Frozen methods, seeds, resampling counts, conditions, and endpoint parameters
`config/safety-envelope.json`	Versioned deterministic actuation constraints
`evidence/dataset-manifest.json`	Source DOI, version, license, selected files, byte sizes, and SHA-256 values
`results/subject-runs.jsonl`	Canonical subject-condition rows with posterior paths and ledger checks
`results/benchmark-summary.json`	Subject-aggregated descriptive results
`results/statistical-tests.csv`	Paired primary-endpoint contrasts and adjusted p-values
`results/safety-cases.csv`	Expected and observed safety-governor outcomes
`paper_values.tex`	Generated LaTeX macros used by article and supplement
`figures/`	Vector quantitative plots and TikZ architecture sources

S3. Dataset Acquisition and Integrity

The acquisition target is the Gait in Neurodegenerative Disease Database version 1.0.0, DOI https://doi.org/10.13026/C27G6C. The downloader first retrieves the publisher's

checksum index. Every selected file is written to a temporary path, hashed, and atomically moved only after its SHA-256 value matches. A run fails on a missing checksum, transport failure, unexpected hash, or incomplete subject record. Raw data are excluded from version control.

The integrity parser enforces unique subject identifiers and maps prefixes to four cohorts: `als` (13), `hunt` (20), `park` (15), and `control` (16), for 64 unique subjects. Each time-series row must contain exactly 13 finite numeric columns. Header sample counts and sampling metadata are parsed separately from the derived stride series. The final manifest records 260 selected source files, their byte sizes, and their checksums.

S4. Complete Feature Definitions

Let L and R denote paired left and right series. The normalized asymmetry functional is

$$\mathrm{Asym}(L,R) = \frac{1}{|V|}\sum_{i\in V}\frac{|L_i - R_i|}{(|L_i| + |R_i|)/2}, \tag{S1}$$

where V excludes positions with denominator at most 10^{-12}. The coefficient of variation uses sample standard deviation and absolute mean. Robust outliers satisfy $|0.67448975(x - \mathrm{median}(x))/\,\mathrm{MAD}(x)| > 4$. Table S2 lists all 25 features.

Table S2. Complete subject-level feature dictionary.

Feature	Family	Definition
`left_stride_mean`	Stride timing	Mean left stride interval
`right_stride_mean`	Stride timing	Mean right stride interval
`bilateral_stride_mean`	Stride timing	Mean of pointwise bilateral stride average
`stride_asymmetry`	Asymmetry	Normalized left–right stride asymmetry
`swing_asymmetry`	Asymmetry	Normalized left–right swing asymmetry
`stance_asymmetry`	Asymmetry	Normalized left–right stance asymmetry
`left_swing_pct_mean`	Stance–swing	Mean left swing percentage
`right_swing_pct_mean`	Stance–swing	Mean right swing percentage
`left_stance_pct_mean`	Stance–swing	Mean left stance percentage
`right_stance_pct_mean`	Stance–swing	Mean right stance percentage
`double_support_mean`	Stance–swing	Mean double-support duration
`double_support_pct_mean`	Stance–swing	Mean double-support percentage
`left_stride_cv`	Variability	Left stride coefficient of variation
`right_stride_cv`	Variability	Right stride coefficient of variation
`left_swing_cv`	Variability	Left swing coefficient of variation
`right_swing_cv`	Variability	Right swing coefficient of variation
`double_support_cv`	Variability	Double-support coefficient of variation
`left_stride_lag1`	Long range	Left stride lag-one Pearson correlation
`right_stride_lag1`	Long range	Right stride lag-one Pearson correlation
`left_stride_dfa`	Long range	Left detrended-fluctuation slope
`right_stride_dfa`	Long range	Right detrended-fluctuation slope
`n_strides`	Signal quality	Number of time-series rows
`recording_duration_s`	Signal quality	Last timestamp minus first timestamp
`missing_fraction`	Signal quality	Missing fraction in source series (zero after strict parse)
`stride_outlier_fraction`	Signal quality	Robust outlier fraction across bilateral stride values

Detrended-fluctuation slopes use non-overlapping windows from the feasible subset of $\{4, 8, 16, 32, 64\}$. Within each window a linear trend is removed; the regression slope of log fluctuation against log scale is returned when at least two scales are available, otherwise zero. All feature outputs must be finite.

S5. Typed Contracts and Ledger Canonicalization

Boundary models are immutable and reject unknown fields. Identifiers must match a restricted lowercase pattern; timestamps must contain a timezone; distributions must sum to one within 10^{-9}; and every numeric input must be finite. A simulated observation is

invalid without a non-negative simulation seed, while an observed or derived observation is invalid if it contains a simulation seed.

An evidence event contains an optional claimed identifier, parent identifier, event type, evidence origin, timestamp, source reference, JSON payload, and schema version. The canonical identifier is

$$h_e = \mathrm{SHA256}(\mathrm{JSON}_{\mathrm{sorted,compact}}(e \setminus \{\texttt{event\_id}\})). \tag{S2}$$

The first event has a null parent; every later parent must equal the preceding event identifier. Verification recomputes every hash and parent relation. Any mismatch raises an integrity error rather than returning a partial replay.

S6. Council Scoring and Deterministic Ranking

For candidate a, current posterior p, and current entropy $H(p)$, the informativeness score is $\mathrm{EIG}(a)/H(p)$ when entropy is positive and zero otherwise. The falsification score selects the two most probable classes, computes the mean absolute class-mean difference divided by pooled standard deviation across the probe's features, and applies $\tanh(d/2)$. The diversity score is $\mathrm{clip}(1 - 0.75k, -1, 1)$ for k previous uses of the family.

The feasibility score is $\mathrm{clip}(1 - c/b - 2m, -1, 1)$, where c is probe cost, b is remaining budget, and m is declared family missingness. It vetoes missing channels or a cost above budget. The safety critic assigns 1.0, 0.5, 0.0, and -0.5 to passive, low, moderate, and high risk after vetoing emergency-stop state, non-permitted risk, or high risk without approval.

The planner validates exactly one score card from each configured critic. Any veto removes the probe. Remaining probes are ordered by equal-weight mean score, then descending EIG, ascending cost, and stable identifier. Ablations remove one predeclared critic without renormalization error because the remaining positive weights are normalized by their sum.

S7. Closed-Loop Reference Algorithm

The complete deterministic procedure is:

1. validate the research case, prior, candidates, sensor availability, risk permissions, and loop configuration;
2. append `run_started` and one `hypothesis_registered` event per class;
3. evaluate confidence, maximum-probe, cost, and emergency-stop rules;
4. construct a planning context from the current posterior, used families, budget, missingness, permissions, and emergency state;
5. obtain council score cards and deterministically select the highest-ranked non-vetoed candidate;
6. append `probe_proposed`; apply the marginal-information rule;
7. execute the deterministic safety governor and append `probe_approved` or `probe_rejected`;
8. for an approval, derive the idempotency key from run, step, and probe; execute the adapter exactly once; append `observation_recorded`;
9. recompute and validate the posterior; append `posterior_updated`; and
10. repeat until a terminal rule, then append `run_stopped`, verify the chain, and return the immutable run.

Replay does not trust stored posterior values. It reconstructs the sequence from observation events and compares the recomputed terminal identifier and stop reason with the recorded run.

S8. Simulator and Safety Envelope

The response simulator is fitted only on explicit training subjects. It rejects duplicate training identifiers and prevents simulation of a subject present in its fit set. For each requested feature, a scenario coefficient is drawn from a predeclared range. Cadence scaling uses coefficients $[-0.9, -0.7]$ applied to the deviation of cadence ratio from one; assistance uses $[-0.25, -0.05]$; and resistance uses $[0.05, 0.25]$. Gaussian noise is limited by both 1% of feature scale and 5% of the class standard deviation. The final value is clipped to within 25% of baseline magnitude. These coefficients are scenario parameters, not estimated treatment effects.

Table S3. Frozen illustrative safety envelope used for deterministic software challenges.

Actuation	Range	Max. duration (s)	Max. step	Max. cumulative (s)
Cadence ratio	0.90–1.10	120	0.05	240
Assistance fraction	0–0.40	120	0.10	180
Resistance fraction	0–0.30	90	0.10	150

All three active actuation kinds require the `clinician_supervision` prerequisite; assistance and resistance are mutually exclusive within a run. Moderate and high risk require explicit approval. Rejection reason codes are stable machine values. The envelope hash is included in every safety decision. The values in Table S3 exist to exercise the implementation and are not a recommendation for any person or device.

S9. Benchmark Conditions and Leakage Controls

Repeated stratified four-fold cross-validation with five repeats yields 20 outer splits. Subjects are sorted before splitting; no subject identifier can occur in both training and test partitions. The benchmark runs 11 conditions for every test subject and repeat, resulting in 3520 frozen rows.

Table S4. Predeclared benchmark conditions.

Method	Scenario	Definition
Fixed	Clean	Stride timing, asymmetry, stance–swing, variability, long range, then quality; stable bundle order
Random	Clean	Seeded random permutation of candidate probes
EIG	Clean	Informativeness critic only
EARL	Clean	Five-critic complete policy
Oracle	Clean	Held-out realized values and exact dynamic programming; non-deployable upper reference
EARL no falsification	Clean	Complete policy without falsification critic
EARL no diversity	Clean	Complete policy without diversity critic
EARL no simulator	Clean	Complete policy without simulator-informed informativeness critic
EARL	Noise	Gaussian noise at 10% of training-fold feature standard deviation
EARL	Missingness	Independent 20% feature removal, retaining at least one feature per probe
EARL	Random feedback	Training-fold cohort labels deterministically permuted

Every run uses a uniform prior and exactly five accepted passive probes. The benchmark disables confidence and marginal-EIG stopping so that all trajectories have equal length. The oracle uses realized held-out values and exact dynamic programming over

remaining probes to maximize the same trapezoidal AUC endpoint. It cannot be deployed and is used only to contextualize headroom.

The configuration digest is `f84ada908ab368602a40a6393078676d1e219c2e1ba1802fc7d6c43201e17124`. It binds the JSON configuration, safety-envelope checksum, and source bytes of the implementation modules that can affect the benchmark.

S10. Endpoints, Aggregation, and Inference

The entropy-reduction AUC integrates $H(p_0) - H(p_t)$ by the trapezoidal rule over the baseline and five post-probe states. Subject-level probabilities and AUC are averaged across the five repeats before descriptive statistics. Macro accuracy is the unweighted mean of per-class accuracies. The reported multiclass Brier score is unscaled, $N^{-1} \sum_i \sum_y (p_{iy} - \mathbf{1}[y_i = y])^2$, using the repeat-averaged probabilities. ECE uses top-label confidence and ten equal-width bins.

The two threshold endpoints use the frozen implementation's distinct aggregation rule. For subject i and repeat r, let T_{ir} be the first accepted probe at which the true-label posterior reaches 0.8, or undefined if it never does. Subject i is counted as reaching if any of its five T_{ir} values is defined. The threshold fraction is the number of such subjects divided by 64. For each reaching subject, compute the median of its defined T_{ir} values, then take the median across reaching subjects. Non-reaching runs are excluded from this conditional count, so a median such as 2.5 is possible. The fraction must not be interpreted as per-run coverage. True labels are available only to retrospective evaluation; operational confidence stopping instead uses the maximum predicted posterior and may stop on an incorrect class.

Mean estimates and paired differences use 10000 percentile bootstrap replicates over the 64 subject aggregates. Two-sided paired sign-flip tests use 100000 Monte Carlo draws because the paired sample exceeds the exact-enumeration threshold. The Monte Carlo p-value uses $(e+1)/(B+1)$. Holm adjustment covers ten complete-EARL contrasts.

Table S5. Frozen primary-endpoint results and complete-EARL paired differences. A negative difference means the comparator has higher AUC.

Comparator	Comparator AUC	EARL minus comparator	Holm p
Fixed	7.230	0.459	0.00010
Random	6.926	0.763	0.00010
EIG	8.010	-0.321	0.00010
Oracle	8.741	-1.052	0.00010
No falsification	8.004	-0.316	0.00010
No diversity	7.693	-0.004	0.66317
No simulator critic	7.214	0.475	0.00010
Missingness	7.343	0.346	0.00010
Noise	7.284	0.405	0.00010
Random feedback	7.022	0.667	0.00010

S11. Deterministic Safety Challenge Cases

The suite contains one valid control and nine boundary/adversarial cases (Table S6). All 10 of 10 outcomes matched expectation.

Table S6. Safety challenge suite. These are software conformance cases, not clinical safety tests.

Case	Expected	Observed	Reason
Valid cadence	Approved	Approved	Within all bounds
Range	Rejected	Rejected	`value_out_of_range`
Duration	Rejected	Rejected	`duration_limit`
Unit	Rejected	Rejected	`unit_mismatch`
Rate	Rejected	Rejected	`rate_limit`
Cumulative	Rejected	Rejected	`cumulative_exposure`
Combination	Rejected	Rejected	Forbidden combination
Prerequisite	Rejected	Rejected	Missing prerequisite
Approval	Requires approval	Requires approval	`human_approval_required`
Emergency stop	Rejected	Rejected	`emergency_stop`

S12. Reproduction Commands and Frozen Checksums

From the package root, the complete workflow is intended to run as follows:

```
uv sync --all-groups
uv run ruff check src tests scripts
uv run ruff format --check src tests scripts
uv run mypy src scripts
uv run pytest --cov=earl --cov-report=term-missing -q
uv run python scripts/fetch_data.py
uv run python scripts/run_benchmark.py --resume
uv run python scripts/generate_figures.py
latexmk -pdf -interaction=nonstopmode -halt-on-error manuscript.tex
latexmk -pdf -interaction=nonstopmode -halt-on-error \
  supplementary-information.tex
uv run python scripts/verify_claims.py
uv run python scripts/build_release.py
```

The definitive result checksums are listed in Table S7. Re-running the benchmark in resume mode produced byte-identical result artifacts before manuscript drafting.

Table S7. SHA-256 checksums of frozen result artifacts.

Artifact	SHA-256
`benchmark-metadata.json`	`99d29f8b3c405bc4bd3c5a80c64c48c30f1b51caf065cbd59018e4edd9a002d0`
`benchmark-summary.json`	`7e7b3b32aa7711d6d202af692ba43602bf3d19ad6383acc2bf3b2eb00f3649dc`
`safety-cases.csv`	`6153e9ece0d035806ec56f8d869c06eb39ab93fe2506a01d171ba29608257a52`
`statistical-tests.csv`	`80d6e94485180cf469d861d5d9236243862af37eb02b0cfb1f875fbaf8e8fa27`
`subject-runs.jsonl`	`3a6b21045c918ce16bcbf025800de53355859e09710ea2559edf956cefca496c`

The release audit must fail if a checksum changes without regeneration, a required condition disappears, a result macro diverges from frozen JSON/CSV, a prohibited clinical claim appears, simulated evidence lacks a label, a citation key is missing, or a LaTeX build leaves unresolved references.

S13. Post Hoc Threshold, Probe-Use, Critic, and Uncertainty Analyses

These analyses were introduced in response to peer review on 9 September 2026. They leave the original 11 conditions, 3520 rows, configuration digest, and confirmatory comparison family unchanged. The implementation is `scripts/revision_analysis.py`; machine-readable outputs are in `results/revision-analysis/`. Each artifact records the appropriate subject, run, selection, or candidate-card denominator.

`secondary-endpoints.csv` contains all 11 conditions, subject and run threshold counts, conditional medians, and subject-bootstrap intervals. Subject attainment is true when any of the five repeated held-out paths reaches the true-label posterior threshold of 0.8. The conditional count first takes each reaching subject's median first-hit count over its reaching paths, then takes the median across reaching subjects. This exactly reuses the frozen aggregation in `earl.statistics.summarize_runs`. The condition is retrospective and label-dependent; it is not the maximum-predicted-posterior stopping condition in Section S14.

`probe-selection-frequency.csv` reports selection counts, paths containing each family, and subjects containing it in any repeat, for all six families and all 11 conditions, including zero counts. The original `probe_order` field stores family names, so those frozen rows alone resolve six families rather than eight bundles. `probe-bundle-selection-frequency.csv` therefore replays all 3520 frozen rows with their original training folds, scenario seeds, fitted models, and policy collaborators, requires each replay to reproduce the frozen family sequence and posterior path exactly, and counts the accepted bundle identifiers; bundle counts must sum to the frozen family counts in every condition, or the audit fails. `record-quality-selection.csv` extracts the signal-quality family without changing its denominators. Stance–swing and variability can each occur twice in a path; family inclusion counts and selection counts are therefore distinct.

The clean-EARL audit refits the original training folds and reproduces all 320 original paths before collecting 1600 decision states. Each critic has 9600 score cards because eight through four remaining candidates are examined across the five steps. `critic-impact.csv` reports local first-choice changes when one critic is removed, with the original posterior, history, candidates, and deterministic tie-break held fixed. `critic-score-variability.csv` reports across-card and within-state score ranges. Feasibility's across-card mean is 0.605 and standard deviation is 0.258; its within-state candidate range is always zero. Safety has mean 1 and standard deviation zero. Neither changes any clean selection, and no critic emits a veto. These are conditional one-step diagnostics, not causal effects or substitutes for the original full-path ablations.

For selected secondary metrics, 10000 bootstrap samples resample subjects within their true cohorts, preserving cohort sizes, after their five final posteriors have been averaged. Models and folds are not refitted. The macro-accuracy 95% intervals are 45.08–66.74% (EARL) and 39.01–59.78% (EIG); Brier intervals are 0.61–0.95 and 0.64–0.99, respectively. The leave-one-subject-out AUC contrast ranges from -0.341 to -0.302, using existing fitted predictions. Neither procedure measures external transportability or removes uncertainty from overlapping training folds. Metadata records seeds, input and source hashes, software versions, and successful path reconstruction.

S14. Post Hoc Heterogeneous-Cost and Early-Stop Scenario

The separate implementation `scripts/revision_scenarios.py` uses the same 64 subjects and 20 outer splits, refitting learned quantities on each training set. It compares three arms: fixed order with five probes, complete EARL with five probes, and complete EARL with a maximum predicted posterior of at least 0.8 as an early stop (at most five probes). All arms have the same candidate set and illustrative costs: stride timing, asymmetry, and signal quality cost one unit per bundle; stance–swing and variability cost two; and long-range dynamics costs three. The 15-unit budget cannot bind within five accepted probes. Confidence is checked at the start of the next loop iteration; true cohort labels never select or stop a probe.

The full configuration, per-run rows, summary, and provenance are stored in `results/revision-scenarios/`. The 960 paths comprise 320 per arm and are not new participants.

Costs change the feasibility ranking, so even the EARL five-probe arm can differ from the original unit-cost condition. Resource counts are means over paths; predictive metrics are computed from each subject's five-repeat averaged final posterior, consistently with the original statistical aggregation. All arms share identical subject–fold keys and all posterior paths pass replay verification.

EARL's early stop changes mean cost from 7.956 to 2.581 illustrative units and mean accepted probes from 5.000 to 1.722. Macro accuracy changes from 55.81% to 48.26%. This scenario illustrates an explicit trade-off under selected assumptions. It does not infer real time or burden savings, model shared acquisition costs, establish a cost-optimal policy, or calibrate the confidence threshold for clinical use.

S15. Post Hoc End-to-End Safety Example and Reproduction

The constructed example uses the first original outer split and the held-out subject `als11` as its baseline; it does not create a new participant. Training-fold statistics fit the likelihood and simulation model. Two low-risk active probes have equal illustrative cost and cadence ratios 1.20 and 1.04. Complete EARL ranks the 1.20 candidate first (aggregate score 0.736 versus 0.635), as does the independent EIG diagnostic ranking (1.590 versus 0.536 bits). The deterministic governor rejects its numeric envelope violation, although planning-time risk declarations alone do not veto it. With continuation enabled, the actual loop removes that candidate, replans, accepts the 1.04 alternative, obtains one simulated observation through `SimulatorAdapter`, and updates the posterior. No rejected action reaches the adapter. The resulting eight-event chain verifies hashes, parent links, and posterior replay. It is a constructed software example, not an estimate of unsafe-action frequency or physical safety.

After checksum-verified acquisition of the original dataset, run the revision analyses separately from the original benchmark:

```
uv run python scripts/revision_analysis.py
uv run python scripts/revision_scenarios.py
uv run python scripts/build_revision_tables.py
make paper
make audit
```

The analysis outputs include artifact hashes and provenance; the scenario verifies the actual selected raw-file bytes against the publisher manifest before loading subjects. The original result checksums in Section S12 must remain unchanged. Generated `revision_values.tex` and `tables/revision-*.tex` carry the separately identified revision values into the manuscript and response. The supplied source archive excludes raw PhysioNet files and local development caches.

S16. Post Hoc Sensitivity Analysis Excluding Record Quality

The implementation is `scripts/revision_sensitivity.py`; outputs are in `results/revision-sensitivity/`. The record-quality bundle is removed from the candidate set before planning, so no clean policy can select it at any step; every other collaborator, including the outer splits, fitted Gaussian models, feature scales, scenario seeds, tie-breaking, and the safety governor, is the original one. The five clean policies are rerun for the 64 subjects and 20 outer splits (1600 rows), each row is replay-verified against its own ledger, and `sensitivity-runs.jsonl` stores the rows in the frozen row format. `sensitivity-summary.json` pairs each policy's frozen summary from `benchmark-summary.json` with the rerun summary computed by the same `earl.statistics` aggregation, together with paired within-subject AUC contrasts (10000 subject-bootstrap replicates), descriptive differences in macro accuracy, Brier score, and ECE, the family distribution of the rerun selections,

and the number of unchanged family paths. Fixed ordering never reaches the sixth family within five probes and is reproduced exactly, which verifies the rerun path. Pairwise EARL-versus-comparator AUC contrasts within the rerun use the original permutation and Holm procedures but form a separate post hoc family. The rerun changes both the evidence available and the selection sequence; it does not identify whether the removed features encode physiology or acquisition protocol, and it does not remove protocol information that other bundles may share.
